# Effect of Optimal Thyroid Replacement Therapy on Chronic Hyponatremia with Focused Review of the Evidence, Mechanisms, and Clinical Implications

**DOI:** 10.7759/cureus.5813

**Published:** 2019-10-01

**Authors:** Nadia Chaudhary, Faiza Warraich, Zabih Warraich, Sami Warraich, Faiz Anwer

**Affiliations:** 1 Internal Medicine, King Edward Medical University, Lahore, PAK; 2 Internal Medicine, McLaren Flint Hospital, Flint, USA; 3 Medicine, United Health Services Wilson Medical Center, Johnson City, USA; 4 Internal Medicine, Sisters of Charity Hospital, Buffalo, USA; 5 Hematology and Oncology, The University of Arizona, Tucson, USA

**Keywords:** chronic hyponatremia, hypothyroidism, thyroid stimulating hormone

## Abstract

We present an unusual case of hyponatremia in an ambulatory hypothyroid patient and review related published literature on PubMed including, original articles, reviews and case reports that describe or refute the association and mechanism for the development of hyponatremia in hypothyroidism.

A 50-year-old female presented in ambulatory clinic with complaints of bilateral hand swelling, fatigue, dizziness, and unsteadiness while walking. Laboratory investigations revealed that she had hypothyroidism and hyponatremia. Thyroid hormone replacement therapy resulted in resolution of hypothyroidism symptoms as well as hyponatremia. A comprehensive search of related literature regarding the development of chronic hyponatremia in hypothyroidism revealed two schools of thought, which we have summarized in this report.

Based on our observations, we conclude that due to overlap in symptoms of hyponatremia and neurological manifestations of hypothyroidism, it is imperative to screen hypothyroid patients for underlying hyponatremia and treat accordingly in order to prevent long-term complications of chronic hyponatremia. Hyponatremia secondary to hypothyroidism resolves with appropriate thyroid hormone replacement therapy, which shows convincing evidence of an association between the two entities.

## Introduction

Hypothyroidism is an endocrine disorder that affects multiple organ systems. Common etiologies for hypothyroidism in adults are an autoimmune disease, pituitary disorders, iodine deficiency, certain medications, and radiation therapy and thyroid surgery. Manifestations of hypothyroidism include fatigue, constipation, cold intolerance, weight gain, hair loss, dry skin, insomnia, and depression, slowed mentation, poor concentration, short term memory impairment, gait abnormalities and bilateral carpal tunnel syndrome [1-3]. Hyponatremia is defined as a serum sodium concentration below 135 mEq/L. The neurologic manifestations of hypothyroidism can be due to the direct effect of lack of thyroid hormone or secondary to hypothyroidism induced hyponatremia. Our literature searches revealed reports that hypothyroidism can cause electrolyte imbalance [4].

Chronic hyponatremia can produce symptoms that are similar to neurologic symptoms of hypothyroidism [5]. Because of symptomatic overlap, mild hyponatremia may remain unnoticed in chronic hypothyroid patients, with serious long-term complications [6]. Hyponatremia has been reported in iatrogenic hypothyroid patients undergoing radioactive iodine therapy (RAI) for thyroid cancer, but it has not been described in ambulatory hypothyroid patients being managed in an outpatient clinic setting [7]. Currently, there are no guidelines to screen hypothyroid patients for hyponatremia. Researchers postulate that causes of hyponatremia secondary to hypothyroidism could be various mechanisms, and it subsides with optimal replacement therapy for hypothyroidism [8].

## Case presentation

A 50-year-old Caucasian female presented to the outpatient clinic with severe pain and swelling of hands bilaterally, extreme fatigue, dizziness while standing from sitting position, irritability, and feeling unsteady while walking. Symptoms had been ongoing for six months. The patient denied any trauma, insect bite on hands, nausea, vomiting, and diarrhea, blood in stools, shortness of breath, heavy menstruation, or altered urinary habits. The patient had a history of diabetes mellitus (DM) type 2 and hypertension (HTN) for seven years, for which she was taking sitagliptin/metformin and amlodipine, respectively.

On examination, the patient was hemodynamically stable. Her hands appeared puffy. There was tingling along the median nerve distribution upon taping the wrists bilaterally. Examination revealed no enlargement of the thyroid gland. The remainder of the physical exam was unremarkable.

On initial presentation with laboratory results, the patient received a diagnosis of subclinical hypothyroidism possibly secondary to autoimmune disease (Hashimoto’s thyroiditis). Laboratory results also showed hyponatremia which was attributed to hyperglycemia secondary to poorly controlled DM. The differential diagnosis for hyponatremia includes hyperlipidemia, adrenal insufficiency, syndrome of inappropriate antidiuretic hormone (SIADH), small cell lung cancer, medications, heart failure, and liver cirrhosis.

Investigations

Laboratory results revealed that her hemoglobin level was 12.6 g/dl, leukocyte count was 8.4×103/µL, platelet count was 254×103/µL, blood sugar level was 207mg/dL, thyroid-stimulating hormone (TSH) level was 20.81 µU/mL (reference range, 0.3 - 5.0 µU/mL) with normal free T3 and T4. Electrolyte panel showed a serum sodium level of 121 mEq/L with normal serum potassium and chloride levels. HbA1c was 8.8%. Renal function tests showed normal serum creatinine and blood urea nitrogen (BUN). Her serum and urine osmolality were also normal. Liver function tests showed normal serum alanine transaminase (ALT) level.

## Discussion

Common etiologies for hyponatremia include diarrhea, heart failure, liver disease, renal disease, adrenal insufficiency, SIADH, and certain drugs. Physicians can classify hyponatremia as acute or chronic. Chronic hyponatremia causes attention deficits, unsteadiness, and increases the risk of falls [5,6]. Clinical manifestations are primarily neurologic and occur due to an osmotic shift of water into brain cells, causing edema [9]. Acute hyponatremia can cause a headache, confusion, irritability, muscle cramps, seizures, and coma. Researchers do not fully understand the mechanism by which deficiency of thyroid hormone causes decreased sodium levels. In the published literature, researchers have proposed a number of mechanisms.

We have summarized our literature search for studies that propose mechanisms for hypothyroidism induced hyponatremia as follows. Bautista et al. (2014) postulated that thyroid hormones affect the expression of electrolyte reabsorption channels on kidney cells thus decreasing the capacity of kidneys to reabsorb electrolytes such as sodium, calcium, potassium, and magnesium [4]. Schmitz et al. (2001) suggested that a decrease in thyroid hormone levels significantly affects the kidney’s ability to reabsorb sodium [8]. Montenegro et al. (1996) proposed that hypothyroidism causes a decrease in the glomerular filtration rate and renal blood flow, which is evident from decreased creatinine clearance in hypothyroid patients [10]. Functional impairment of kidneys leads to hyponatremia [11]. Kohno et al. (1986) suggested that hypothyroidism leads to atrial natriuretic peptide (ANP) deficiency, precipitating free water retention, and consequently, hyponatremia [11]. Liamis et al. (2017) suggested that chronic hypothyroidism decreases cardiac output, triggering antidiuretic hormone (ADH) release, which causes water retention and consequently, hyponatremia [12]. We have summarized the probable mechanisms for hypothyroidism induced hyponatremia described in the literature in Table 1.

**Table 1:** Summary of proposed mechanisms for development of hyponatremia secondary to hypothyroidism GFR- glomerular filtration rate; RBF- renal blood flow; ANP- atrial natriuretic peptide; ADH- antidiuretic hormone

AUTHOR	PROPOSED MECHANISM	STUDY TYPE
Bautista et al., 2014 [4]	Decreased expression of Na reabsorption pumps on renal tubule in hypothyroidism leads to decreased sodium reabsorption thus precipitating hyponatremia.	Case Report
Montenegro et al., 1996 [10]	Decreased GFR and RBF leads to decreased excretion of free water that brings on hyponatremia.	Retrospective review
Kohno et al., 1986 [11]	Decreased systemic release of ANP causes water retention that eventually brings on hyponatremia.	Rodent model
Liamis et al., 2017 [12]	Decreased Cardiac output secondary to hypothyroidism prompts increased ADH release that causes water retention and consequently hyponatremia.	Retrospective review
Schmitt R et al., 2002 [13]	Hypothyroidism induced renal changes cause hypo-osmolar hyponatremia.	Case Report

There is considerable debate in the medical community about the potential link of hyponatremia with hypothyroidism. Review of literature revealed that while some studies support the theory of hypothyroidism-induced hyponatremia others refute it. Leroith et al. (1980) investigated hyponatremia in a patient with primary hypothyroidism and adrenocorticotropic hormone (ACTH) deficiency. The physician corrected sodium levels in this patient with salt and hydrocortisone replacement therapy, which showed no link of hyponatremia with hypothyroidism [14]. Sun et al. (2012) carried out a retrospective review in chronic hypothyroid patients and concluded that in non-hospitalized, ambulatory patients even severe deficiency of thyroid hormone does not cause significant hyponatremia [15]. A retrospective study by Berndt et al. (2015) for investigating any possible link of hyponatremia in hypothyroid patients showed that deficiency of thyroid hormone had no effect on hyponatremia occurrence [16]. Another retrospective analysis by Wolf et al. (2017) showed that decreased sodium levels in hypothyroid patients were often secondary to other medical conditions [17]. A prospective study by Hammani et al. (2013) in thyroid cancer patients concluded that there was no association between hyponatremia and hypothyroidism [18].

The probable cause of hyponatremia in our patient is hypothyroidism, as her serum sodium level improved after appropriate levothyroxine dosage and adjustment. Hyponatremia can be secondary to uncontrolled DM or hyperlipidemia [19]. The physician treated both of these conditions appropriately in our patient.

Mild hyponatremia can go unnoticed in hypothyroid patients managed in the ambulatory setting and can result in long term complications of attention deficits, unsteadiness, increased risk of fracture, and osteoporosis [5,6]. Therefore, it is imperative to screen chronic hypothyroid patients for electrolyte abnormalities, specifically hyponatremia. Hypothyroidism-induced hyponatremia is managed by correcting the underlying thyroid deficiency.

**Figure 1:**
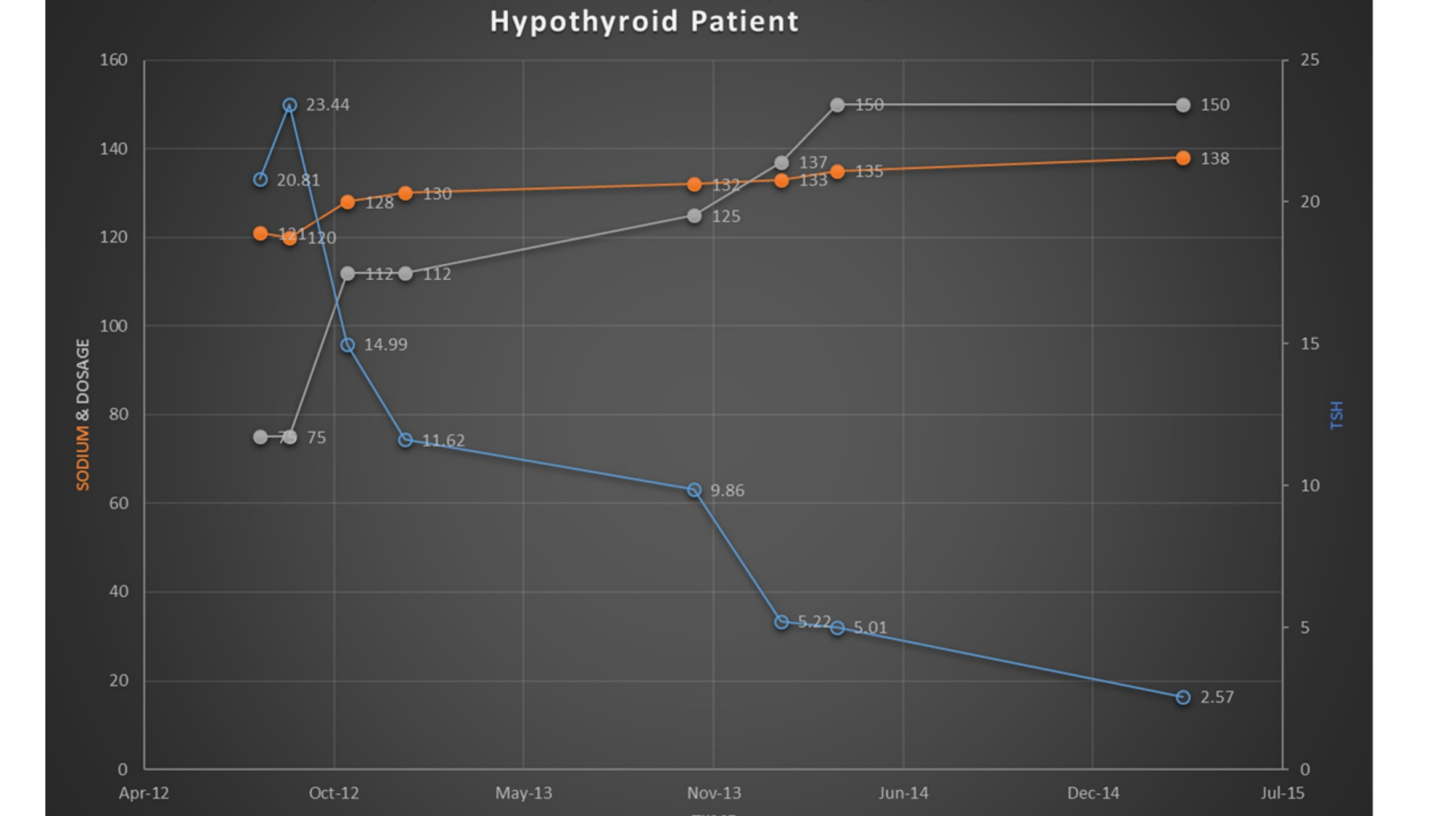
Effect of thyroid replacement therapy on hyponatremia in a hypothyroid patient

Figure 1 depicts the effect of optimal thyroid hormone replacement therapy on serum sodium and TSH levels in our patient.

## Conclusions

Serum sodium level improved only after optimal replacement therapy with Levothyroxine, which confirms the relationship of hyponatremia to thyroid hormone deficiency in this patient. Her hyponatremia was initially thought to be secondary to hyperglycemia and/or hypertriglyceridemia. However, hyponatremia persisted even after serum triglyceride and HbA1c levels became normal. Her sodium level normalized after appropriate thyroxine replacement therapy. Therefore, it is recommended that physicians should always screen chronic hypothyroid patients for electrolyte abnormalities to prevent complications.
